# Detection of Neural Activity in the Brains of Japanese Honeybee Workers during the Formation of a “Hot Defensive Bee Ball”

**DOI:** 10.1371/journal.pone.0032902

**Published:** 2012-03-14

**Authors:** Atsushi Ugajin, Taketoshi Kiya, Takekazu Kunieda, Masato Ono, Tadaharu Yoshida, Takeo Kubo

**Affiliations:** 1 Department of Biological Sciences, Graduate School of Science, The University of Tokyo, Bunkyo-ku, Tokyo, Japan; 2 Division of Life Sciences, Graduate School of Natural Science and Technology, Kanazawa University, Kanazawa, Japan; 3 Laboratory of Applied Entomology and Zoology, Graduate School of Agriculture, Tamagawa University, Machida, Japan; 4 Honeybee Science Research Center, Tamagawa University, Machida, Japan; University of Arizona, United States of America

## Abstract

Anti-predator behaviors are essential to survival for most animals. The neural bases of such behaviors, however, remain largely unknown. Although honeybees commonly use their stingers to counterattack predators, the Japanese honeybee (*Apis cerana japonica*) uses a different strategy to fight against the giant hornet (*Vespa mandarinia japonica*). Instead of stinging the hornet, Japanese honeybees form a “hot defensive bee ball” by surrounding the hornet *en masse*, killing it with heat. The European honeybee (*A. mellifera ligustica*), on the other hand, does not exhibit this behavior, and their colonies are often destroyed by a hornet attack. In the present study, we attempted to analyze the neural basis of this behavior by mapping the active brain regions of Japanese honeybee workers during the formation of a hot defensive bee ball. First, we identified an *A. cerana* homolog (*Acks* = *Apis cerana kakusei*) of *kakusei*, an immediate early gene that we previously identified from *A. mellifera*, and showed that *Acks* has characteristics similar to *kakusei* and can be used to visualize active brain regions in *A. cerana*. Using *Acks* as a neural activity marker, we demonstrated that neural activity in the mushroom bodies, especially in Class II Kenyon cells, one subtype of mushroom body intrinsic neurons, and a restricted area between the dorsal lobes and the optic lobes was increased in the brains of Japanese honeybee workers involved in the formation of a hot defensive bee ball. In addition, workers exposed to 46°C heat also exhibited *Acks* expression patterns similar to those observed in the brains of workers involved in the formation of a hot defensive bee ball, suggesting that the neural activity observed in the brains of workers involved in the hot defensive bee ball mainly reflects thermal stimuli processing.

## Introduction

In nature, animals threatened by predators exhibit a variety of adaptive behaviors to escape or actively defend themselves against the predators [1]. Some animals exhibit characteristic anti-predator behaviors against their natural enemies that are considered to be an evolutionary consequence of adaptation to the threat of natural enemies [2], [3]. The neural bases of the anti-predator behaviors and how during evolution they were acquired, however, remain unknown.

Honeybees (Genus *Apis*) commonly use their stingers to counterattack an intruder [4]. Japanese honeybees (*Apis cerana japonica*), however, fight against the giant hornet (*Vespa mandarinia japonica*), their most formidable natural enemy [5], by exhibiting a characteristic behavior called ‘hot defensive bee ball formation’.

In autumn, giant hornets attack Japanese honeybee colonies to steal their larvae and pupae. If a foraging hornet tries to enter the beehive, a group of more than 500 workers quickly forms a spherical assemblage called a ‘hot defensive bee ball’, trapping the hornet inside the ball. In the ball formation, honeybees vibrate their flight muscles to produce heat. The temperature in the ball quickly rises to almost 47°C, which is lethal to the hornet but not to the honeybees. The high temperature phase continues for approximately 20 min. Within ∼30 to 60 min after initiating the bee ball formation, the hornet is killed by the heat produced [6]. On the other hand, European honeybees (*A. mellifera ligustica*), which are a related but allopatric species and were introduced to Japan in the Meiji era (about 140 years ago) for apiculture, exhibit only stinging behavior against the hornet. The rigid exoskelton of the giant hornet renders the bee stings ineffective, however, and colonies of the European honeybees are often destroyed [7]. Thus, the defensive bee ball formation is considered to result from Japanese honeybee-specific selective pressure to avoid predation by the giant hornets that inhabit East Asia, including Japan [6].

We used the Japanese honeybee's defensive behavior as a model of the evolution of adaptive anti-predator behavior against a natural enemy. In the present study, to elucidate the neural mechanism underlying this behavior, we used an immediate early gene (IEG) to map the active brain regions of Japanese honeybee workers during the formation of a defensive bee ball. IEGs are widely utilized for neuroethological studies in vertebrates [8]–[12], and we recently identified the first insect neuronal IEG from the European honeybee, naming it *kakusei* [13]. The *kakusei* transcript does not contain a long open reading frame (ORF) that encodes a protein, suggesting that it functions as a non-coding RNA, unlike vertebrate IEGs, which generally encode proteins [10]. In a previous study, we used *kakusei* as a neural activation marker to identify the brain regions in the European honeybee that are active during foraging behavior [13], [14]. In the present study, we identified the *kakusei* homolog in the Japanese honeybee and found that the neural activity of one subtype of intrinsic neurons of the mushroom bodies (MBs) is preferentially increased in the brains of the Japanese honeybee workers during the formation of a hot defensive bee ball. Furthermore, we asked what kind of sensory information induces the pattern of neural activity observed during defensive bee ball formation and found that it may relate to the processing of thermal information.

## Results

### Identification of the *kakusei* homolog in the Japanese honeybee

**Figure 1:**
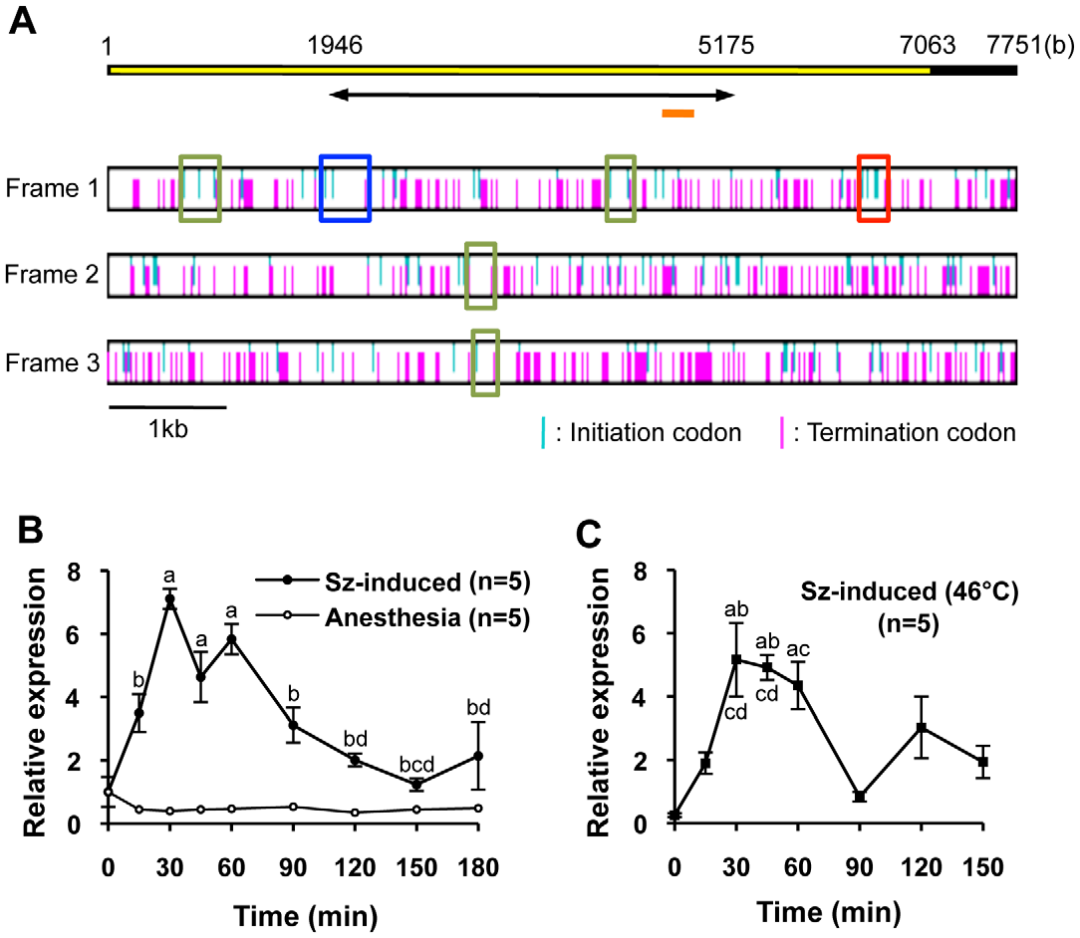
Identification and characterization of *Acks*, the Japanese honeybee *kakusei* homolog, as a non-coding IEG. (A) Overview of *Acks* cDNA and open reading frame (ORF) analysis. The yellow bar and black bar represent the *Acks* cDNA region highly conserved among the *Acks* and *kakusei* cDNAs and the region adjacent to the *Acks* cDNA, respectively. The arrow indicates the region corresponding to the putative neural activity-inducible *Acks* transcript. The orange bar indicates the region corresponding to sense and antisense probes used in the *in situ* hybridization. Horizontal boxes under the upper yellow and black bar indicate open reading frame analysis in each reading frame of the *Acks* cDNA, respectively. The blue and pink bars present in each box indicate positions of initiation and termination codons, respectively. Colored squares on the horizontal boxes indicate potential ORFs longer than 150b. The blue (in Frame 1) and red box (in Frame 1) indicate the longest ORF and the ORF conserved among *Acks* and *kakusei* cDNAs, respectively. (B) Time course of *Acks* expression level investigated by quantitative RT-PCR after seizure induction under room temperature (25°C). Values are means ± SEM (a, different from 0 min *P*<0.01; b, different from 30 min *P*<0.01; c, different from 45 min *P*<0.01; d, different from 60 min *P*<0.01; Tukey-Kramer's test). Sz-induced, seizure-induced. (C) Time course of *Acks* expression level investigated by quantitative RT-PCR after seizure induction under the high temperature (46°C). Values are means ± SEM (a, different from 0 min *P*<0.01; b, different from 15 min *P*<0.05; c, different from 90 min *P*<0.01; d, different from 150 min *P*<0.05; Tukey-Kramer's test).

To map the active regions in the brains of the Japanese honeybee workers during the formation of a defensive bee ball, we intended to use the *kakusei* homolog as a neural activity marker. Because no *kakusei* homolog had been identified in any other animal species, including insects, however, we first amplified parts of *kakusei* cDNA from the Japanese honeybee using primers designed from the European honeybee *kakusei* cDNA sequences. Rapid amplification of the cDNA end (RACE) method was then used to identify the *Apis cerana kakusei* (*Acks*: *Apis cerana kaku*s*ei*) cDNA of approximately 7.8 kb in length. No 5′-upstream or 3′-downstream cDNA sequence was obtained by the RACE method, leading us to conclude that we obtained a full-length *Acks* cDNA. The nucleotide sequences of *Acks* cDNA of approximately 7.1 kb in length shared approximately 85% sequence identity with the European honeybee *kakusei* cDNA, and had additional unique sequences of approximately 700b at its 3′ end. The *Acks* cDNA contained six putative ORFs longer than 150b, and the longest ORF was 342b (Figure 1A). A comparison of the positions of the ORFs with those of *kakusei* cDNA revealed that one of the six ORFs is conserved among *kakusei* and *Acks*. This conserved ORF, however, was located at the 3′-region of the *Acks* cDNA, and its length was only 183b (Figure 1A). We previously showed that, in addition to the neural activity-inducible *kakusei* transcript, multiple neural activity-independent transcripts are constitutively expressed from the same *kakusei* locus, and the nucleotide sequences corresponding to +1925b to +5160b of the consensus *kakusei* cDNA are specifically contained in the neural activity-inducible *kakusei*-transcript [15]. Comparison of the structure and nucleotide sequences of *Acks* and *kakusei* cDNAs revealed that the nucleotide sequences corresponding to +1946b to +5175b of *Acks* cDNA was equivalent to the *kakusei* cDNA corresponding to the neural activity-inducible transcript (Figure 1A). These findings suggest that the cloned *Acks* cDNA also contained nucleotide sequences corresponding to the putative neural activity-inducible *Acks* transcript, and that, like the *kakusei-*transcript, the *Acks* transcript functions as a non-coding RNA.

### Transient induction of *Acks* expression in response to seizures

In our previous study, quantitative reverse transcription-polymerase chain reaction (RT-PCR) revealed that *kakusei* expression is induced transiently in the worker brain, peaking at 15 min after a seizure induced by awakening from anesthesia [13]. To determine whether *Acks* has properties as a neural IEG, we used quantitative RT-PCR to analyze the time course of *Acks* expression in response to seizures. The primers and probes for quantitative RT-PCR were designed based on the nucleotide sequences corresponding to the above-mentioned putative neural activity-inducible *Acks* transcript (Figure 1A). We induced seizure by awakening the Japanese honeybee workers from CO_2_-induced anesthesia at room temperature (25°C) as described previously [13], and measured the *Acks* expression levels in the whole brain using quantitative RT-PCR. The relative *Acks* expression was approximately 5 to 7-fold higher from 30 to 60 min after seizure induction, and then decreased to basal levels (Figure 1B). In control bees kept under anesthesia, there was no significant change in the expression level at any time-point sampled. This finding suggested that *Acks* shares similar properties with the European honeybee neural IEG, *kakusei*. In addition, we also examined whether *Acks* upregulation after neural activation begins earlier under a higher temperature, because the temperature in the hot defensive bee ball rises to almost 47°C [6], [16]. In the bees awakened from anesthesia under the high temperature (46°C), *Acks* expression also peaked at 30 to 60 min (Figure 1C). Although there was an additional expression peak at 120 min, there was no statistically significant difference between the basal *Acks* expression levels and expression at 120 min. The expression levels during peak times at the higher temperature were similar to those at room temperature. The *ef-1α* (*elongation factor-1 alpha*) expression levels did not differ significantly between control and seizure-induced workers at any sampling time, irrespective of the experimental temperature (25°C or 46°C; data not shown). Based on these findings, we concluded that *Acks* has neural IEG properties, even at the high temperature the bees are exposed to during the formation of a hot defensive bee ball.

### Visualizing neural activity in the Japanese honeybee brain with detection of *Acks* expression

**Figure 2:**
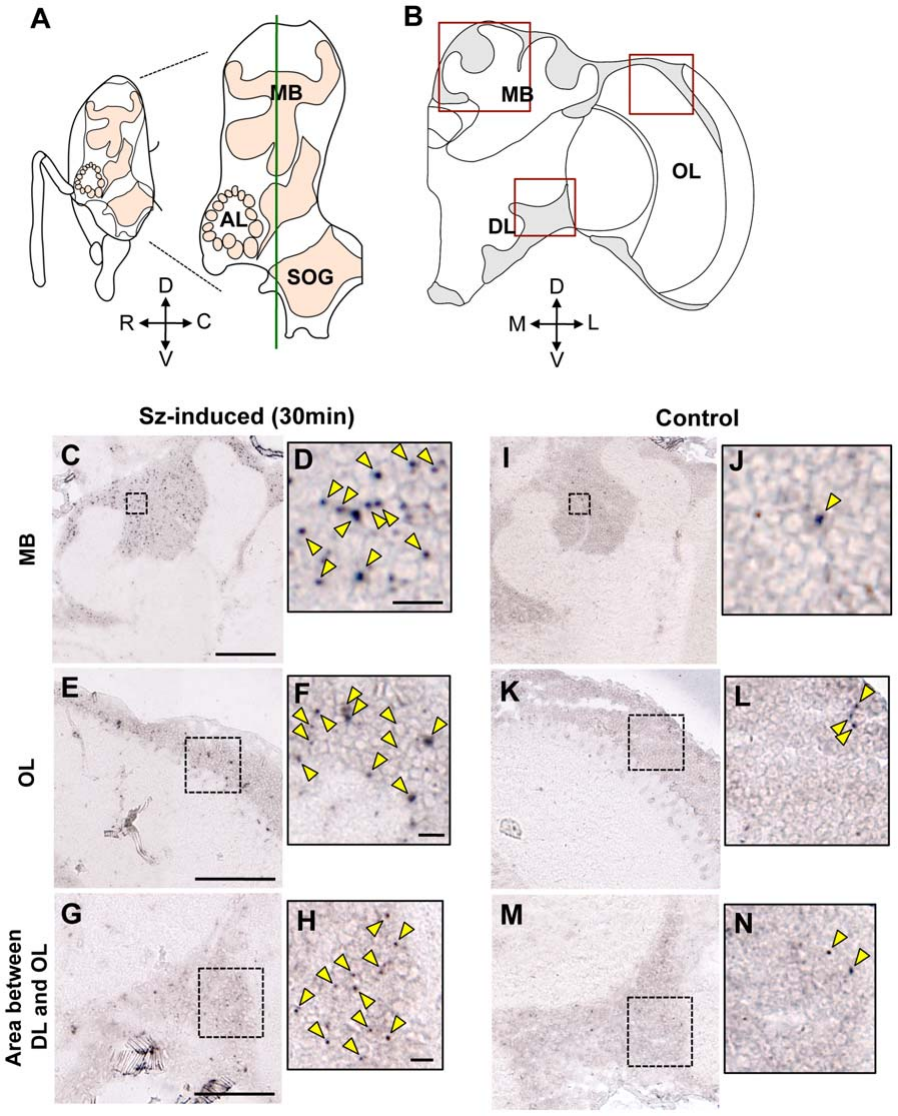
Seizure-induced neural activity in the brains of the Japanese honeybee workers. (A) Schematic diagram of the lateral view of a bee brain. Areas colored in light orange indicate neuropil regions. D, dorsal; V, ventral; R, rostral; C, caudal. MB, mushroom bodies; AL, antennal lobes; and SOG, subesophageal ganglion. The green line indicates the position of sections analyzed in this experiment. (B) A schematic diagram of a middle right brain hemisphere of the Japanese worker honeybee. Areas colored in light grey indicate brain areas where the somata of neurons are located. Red squares correspond to brain areas whose *in situ* hybridization results are presented below. M, medial; L, lateral. OL, optic lobe; DL, dorsal lobe. (C–N) Expression analysis of *Acks* by *in situ* hybridization using coronal brain sections of seizure-induced (Sz-induced) (C–H) or control Japanese honeybee workers (I–N). The upper panels (C and I), middle panels (E and K), and lower panels (G and M) correspond to MB, OL, and area between the DL and OL, which are boxed in (B). Bars indicate 100 µm. (D, F, H, J, L, and N) Magnified views of the regions delineated by dotted lines in panels (C), (E), (G), (I), (K), and (M), respectively. Yellow arrowheads indicate *Acks* signals. Bars indicate 10 µm.

We then examined whether *Acks* can be used as a neural activity marker in the Japanese honeybee brain by visualizing the spatial distribution of the *Acks* transcript in the brain after seizure induction using *in situ* hybridization with digoxigenin (DIG)-labeled antisense probe based on the putative neural activity-inducible *Acks* transcript (Figure 1A). Bees at 30 min after seizure induction and control bees without anesthesia or seizures were analyzed. In the seizure-induced bees, a large number of *Acks* signals was observed in most brain regions, including the MBs (higher brain center), optic lobes (OLs, primary visual center), and a restricted area between the dorsal lobes (DLs) and the OLs, as spots localized in the somata (Figure 2C–H). A previous study using fluorescent *in situ* hybridization demonstrated that *kakusei* transcript signals are localized exclusively in the nuclei, suggesting that the *kakusei* transcript functions as a nuclear noncoding RNA [13]. Thus, our findings of the spotted *Acks* signals in the somata coincided with our notion that the *Acks* transcript also functions as a nuclear noncoding RNA. On the other hand, the brains of the control bees contained very few signals (Figure 2I–N). These findings suggest that neural activity was induced in various brain regions, including the MBs, OLs, and the area between the DLs and the OLs, in the Japanese honeybees soon after induction of the seizure. A time course analysis demonstrated that *Acks* expression peaked at 30 and 60 min after the seizure (Figure 1B and C). Thus, we concluded that *Acks* could be utilized as a marker of neural activity that occurred 30 to 60 min before in the brains of Japanese honeybees, although we could not detect the spotted signal in the antennal lobes (ALs, primary olfactory center), suggesting that *Acks*, like *kakusei*, is not useful for detecting neural activity in the ALs (data not shown).

### Identification of brain regions active in the Japanese honeybees during the formation of a hot defensive bee ball

**Figure 3:**
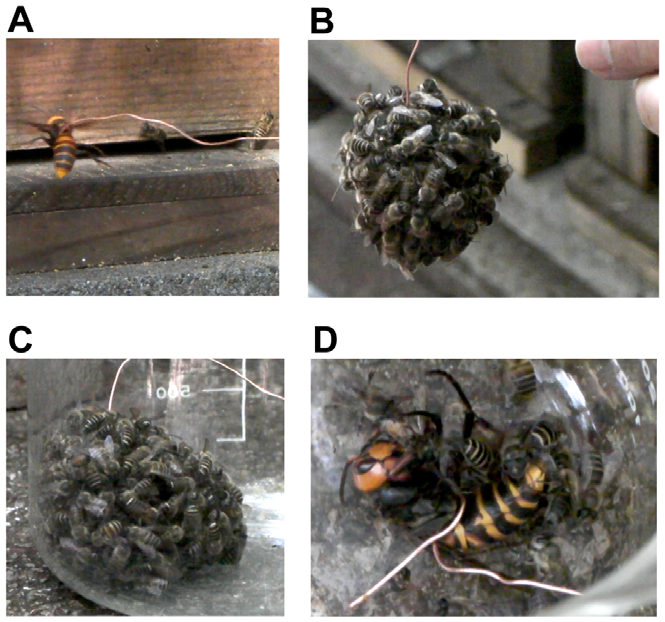
Sampling of workers from an artificially formed hot defensive bee ball. (A) Presentation of a wire-hung hornet to the beehive as a decoy. (B) Hundreds of workers form a hot defensive bee ball surrounding the wire-hung giant hornet. (C) Bee ball recovered in a glass beaker. (D) The giant hornet is dead 60 min after the bee ball forms.

Using *Acks* as the neural activity marker, we next attempted to find brain regions of the Japanese honeybee workers that were active during the formation of a hot defensive bee ball. Because the hot defensive bee ball is usually formed within the beehive [6], it is difficult to collect only the workers involved in forming the bee ball. Therefore, we attempted to induce Japanese honeybee workers to form a bee ball artificially using giant hornets as a decoy. First, the bees were presented with a giant hornet that was hung by a wire at the entrance of the beehive (Figure 3A). After a short time (approximately 5 min), when the decoy giant hornet was inserted into the beehive through the entrance, the Japanese honeybee workers immediately crowded around the decoy hornet to form a hot defensive bee ball (Figure 3B). The bee ball was then recovered from the upper site of the hive and transferred to a glass beaker to collect only workers that formed the bee ball (Figure 3C). We sampled the bees from the surface of the bee ball at 0, 30, and 60 min after formation of the bee ball and examined the *Acks*-expressing brain regions using *in situ* hybridization (n = 5, 7 and 7 for 0, 30 and 60 min, respectively). The decoy hornet inside the bee ball was dead within 60 min after the formation of the bee ball, as usually occurs in nature (Figure 3D).

**Figure 4:**
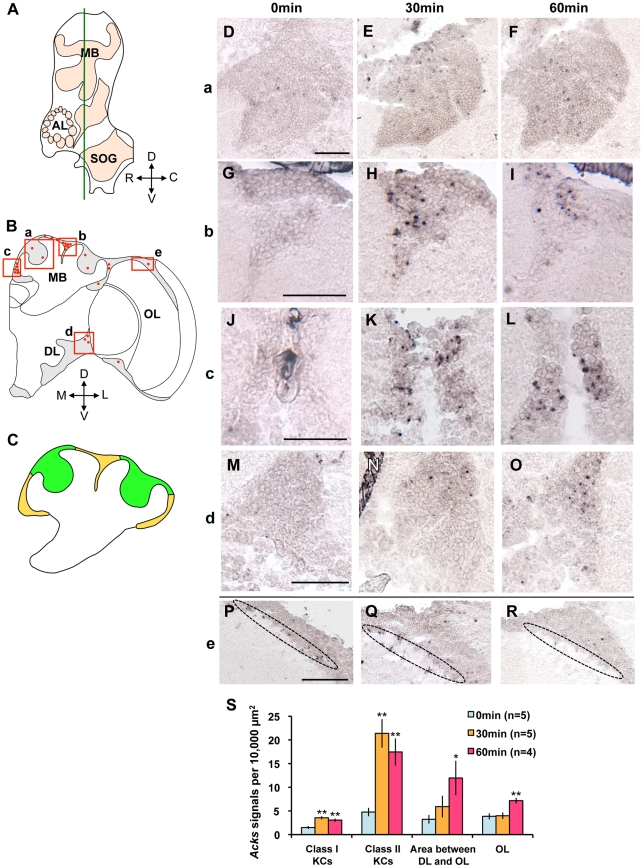
Neural activity in the middle part of the brain during bee ball formation. (A) Schematic diagram of the lateral view of a bee brain. The green line indicates the position of sections that correspond to a middle part of the brain used for this *in situ* hybridization experiment. (B) Schematic representation of the *Acks* signals detected in the right brain hemisphere of the workers that formed the bee ball. The red dots indicate induced *Acks* signals at 30 or 60 min after the bee ball formation. The boxed regions (a–e) correspond to the Class I KCs whose somata are located inside the calyces (a), parts of the Class II KCs whose somata are located outside of calyces (b and c), the restricted area located between the DL and lobula of the OL (d), and a part of the OL (e), whose *in situ* hybridization results are presented in the right panels (D–R). (C) Magnified schematic representation of the MB indicating the distribution of the somata of the Class I (green) and the Class II KCs (yellow), respectively. (D–R) *In situ* hybridization of *Acks* in each brain area shown in (B) in the brains of workers at 0 (D, G, J, M, and P), 30 (E, H, K, N, and Q) and 60 min (F, I, L, O, and R) after the bee ball formation. (D–F), (G–I), (J–L), (M–O), and (P–R) correspond to the boxed brain regions (a), (b), (c), (d), and (e), respectively. The dotted *Acks* signals were detected most densely in the Class II KCs (H, I, K, and L), and less densely in the Class I KCs (E and F) at 30 and 60 min after the bee ball formation, respectively. Note that the *Acks* signals were detected moderately in the restricted region between the DLs and the lobula of the OLs (O), and less densely in the OLs (R) at 60 min after the bee ball formation. Staining observed in area surrounded by dotted ellipse (P–R) represents non-specific staining of trachea, which was also observed in sections hybridized with the sense probe (data not shown). Bars indicate 50 µm. (S) Quantification of *Acks*-positive cells in various brain regions. Values are means ± SEM. Asterisks indicate significant difference compared to that at 0 min (*, *P*<0.05; **, *P*<0.01; Dunnett's test).

The previous experiments indicated a 30- to 60-min time lag between neural activation and *Acks* upregulation in the worker brain. Therefore, we considered that the *Acks* signals detected at each sampling time mainly reflect neural activity involved in the following phases of the bee ball formation; 0 min: normal phase before presentation of the hornet; 30 min: early phase of bee ball formation, such as the recognition of the hornet and/or formation of the bee ball; 60 min: late phase of the bee ball formation, such as the maintenance of the bee ball. In several brain regions, characteristic *Acks* expression was observed in a region-preferential and sampling time-dependent manner (Figure 4B).

Honeybee MBs are a paired structure comprising many thousands of intrinsic neurons called Kenyon cells (KCs). The KC dendrites form two cup-shaped neuropils called calyces and terminals of KC axons and their postsynaptic targets form the lobes. The KCs are classified into two distinct types, termed Class I and Class II KCs [17]–[19]. Whereas somata of the Class I KCs are located inside of the MB calyces, somata of the Class II KCs are located outside of the calyces (Figure 4C). When *in situ* hybridization was performed with sections corresponding to the middle (Figure 4, Figure S1, and Figure S2), more rostral (Figure S3) and more caudal brain parts (Figure S4), the *Acks* signals were most densely detected in the Class II KCs at 30 and 60 min after the bee ball formation (Figure 4H, I, K, and L, Figure S3H, I, K, and L, and Figure S4H, I, K, and L), whereas they were scarcely detected at 0 min (Figure 4G and J, Figure S3G and J, and Figure S4G and J). In contrast, much less *Acks* signals were detected in the OLs, during sampling time (Figure 4P–R, Figure S3M–O, and Figure S4M–O). In the middle brain part, however, *Acks* signals were also detected in some neurons whose somata formed a cluster in a restricted region between the DLs and the lobula of the OLs at 60 min after the bee ball formation (Figure 4O and Figure S2). Scarce *Acks* signals were detected at 0 min in this restricted region between the DLs and the lobula of the OLs (Figure 4M), indicating that the *Acks* signals were detected in a sampling time-dependent manner. No significant signal was detected in any of the brain regions hybridized with sense probe (data not shown).

Statistical analyses of the number of *Acks* signals detected in *in situ* hybridization experiments using sections corresponding to the middle brain parts (n = 5, 5, and 4 for 0, 30, and 60 min, respectively) revealed that there was a significant increase in the number of *Acks* signals at 30 and 60 min after the bee ball formation in both the Class I and the Class II KCs, and at 60 min after the bee ball formation in the brain area between the DLs and the OLs, and in the OLs (Figure 4S). The *Acks* signal density, however, was highest in the Class II KCs at both 30 and 60 min after the bee ball formation. These findings suggested that neural activity of the KCs, especially the Class II KCs and a certain subpopulation of neurons located between the DLs and the OLs, was increased in the brains of workers during formation of the hot defensive bee ball. It is also plausible that some visual input was processed in the OLs of the workers after the bee ball formation, because we picked up the bee ball outside the hives to sample the workers.

### Comparison with brain regions active in the Japanese honeybee workers that are exposed to heat or an alarm pheromone component

**Figure 5:**
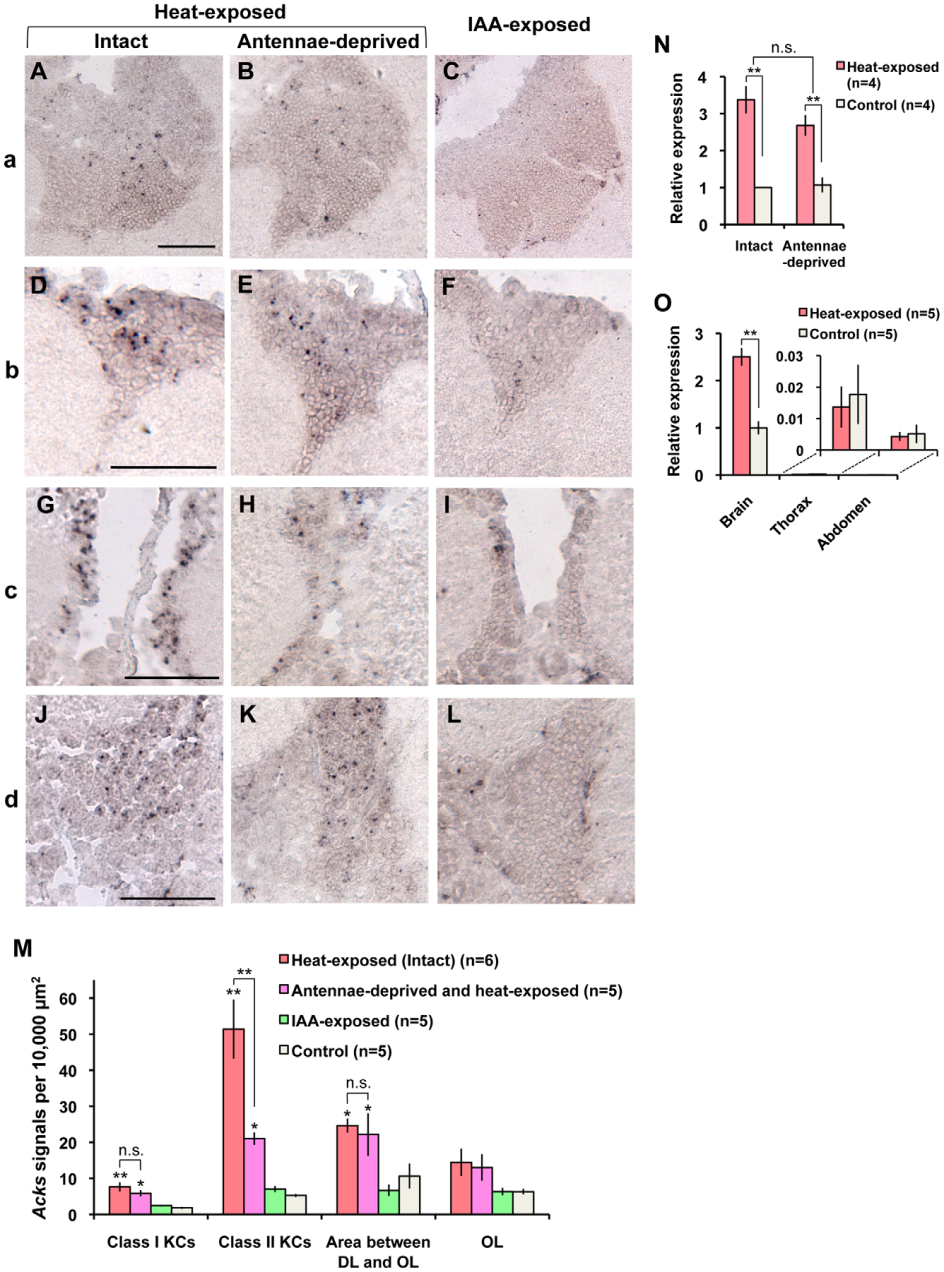
Neural activity in the brains of workers exposed to high temperature or IAA. (A–L) *In situ* hybridization of *Acks* in each brain area shown as Figure 4B in the brains of workers exposed to 46°C heat (A, D, G, and J), workers whose antennae are deprived before heat-exposure (B, E, H, and K), and workers exposed to IAA (C, F, I, and L). (A–C), (D–F), (G–I), and (J–L) correspond to the boxed brain regions shown as in Figure 4B (a), (b), (c), and (d), respectively. In the brains of heat-exposed bees, the dotted *Acks* signals were detected most densely in the Class II KCs (D and G) and moderately in the restricted area between the DLs and OLs (J), and much less densely in the Class I KCs (A), whereas there was some decrease in the *Acks* signals in the Class II KCs of antennae-deprived and heat-exposed workers (E and H). On the other hand, scarce or no significant signals were detected in these brain regions in IAA-exposed workers (C, F, I, and L). Bars indicate 50 µm. (M) Quantification of *Acks*-positive cells in various brain regions. Values are means ± SEM. Asterisks indicate significant difference compared to control (*, *P*<0.05; **, *P*<0.01; Dunnett's test). Student's *t*-test was used to compare heat-exposed intact and heat-exposed antennae-deprived workers (n.s. = non-significant; **, *P*<0.01). (N) The results of quantitative RT-PCR showing the *Acks* expression in the MBs of intact and antennae-deprived workers under usual (33°C) and high (46°C) temperature. Each experimental group contained four lots of workers. A two-way ANOVA revealed that there was no interaction between temperature and ablation of antennae (n.s. = non-significant, *P* = 0.15), and then Student's *t*-test was conducted for intergroup comparison (**, *P*<0.01). Values are means ± SEM. (O) The results of quantitative RT-PCR showing *Acks* expression in the brain, thorax, and abdomen of heat-exposed intact workers. Asterisks indicate a significant difference between heat-exposed and control workers within the same tissues (**, *P*<0.01; Student's *t*-test). The results for the thorax and abdomen are shown in the magnified graph because these values were extremely low. Values are means ± SEM.

During the formation of a hot defensive bee ball, the Japanese honeybee workers are exposed to high temperature typically around 47°C [6]. In addition, isoamyl acetate (IAA), which is the major component of the honeybee alarm pheromone [20], is emitted from a hot defensive bee ball [6]. Therefore, to investigate what kind of information processing induces the observed neural activity in the brains of bees from the defensive bee ball, we examined *Acks* expression patterns in the brains of Japanese honeybee workers exposed to 46°C heat or IAA. In the brains of the workers exposed to high temperature (46°C) for 30 min, a lot of spotted *Acks* signals were preferentially detected in the Class II KCs (Figure 5D and G) and sparsely distributed in the Class I KCs (Figure 5A). *Acks* signals were also detected in some neurons whose somata formed a cluster in a restricted region between the DLs and the lobula of the OLs (Figure 5J).

Statistical analyses of the number of *Acks* signals detected in *in situ* hybridization experiments using sections of brains of heat-exposed (n = 6) and non-treated control workers (n = 5) indicated that the numbers of *Acks* signals significantly increased in the Class I and Class II KCs and in the brain area between the DLs and the OLs in the heat-exposed workers compared with those of control workers, whereas there were no significant differences of the number of *Acks* signals in the OLs (Figure 5M). The highest *Acks* signal density, however, was observed in the Class II KCs. This *Acks* signal distribution pattern in the brains of workers exposed to heat well resembles that observed in the brains of workers involved in the formation of the hot defensive bee ball (Figure 4, Figure S3, and Figure S4).

A previous study reported that ablation of the antennal flagella impaired warmth avoidance behavior in the European worker honeybees [21]. Therefore, we next evaluated the contribution of sensory input from the antennae to the activity of brain neurons under high temperature (46°C) (Figure 5B, E, H and K). Statistical analyses revealed that the density of *Acks* signals detected in the Class II KCs of heat-exposed workers whose two antennae were ablated at their bases was significantly lower than that in heat-exposed intact workers (Figure 5M). There was no significant difference, however, in the *Acks* signal density in the Class I KCs, in the areas between the DLs and the OLs, or in the OLs between the intact and antennae-deprived workers (Figure 5M). These results suggested that thermal information received by the antennae is mainly conveyed to the Class II KCs and that the MBs receive thermal sensory input from other body parts besides the antennae. On the contrary, quantitative RT-PCR using the RNAs extracted from the MBs revealed that the difference in the *Acks* expression level in the MBs between the antennae-deprived and intact workers under high temperature was not statistically significant. Antennae-deprived workers, however, exhibited a tendency toward a 20% decrease in the induced *Acks* expression level compared with the intact workers (Figure 5N). This result suggested that contribution of the antennal sensory input to the neural activity in the whole MBs of heat-exposed workers is partial at most.

In contrast, no significant increase in the density of *Acks* signal was detected in the MBs (Figure 5C, F and I) or the restricted region between the DLs and the OLs (Figure 5L) in the brains of workers exposed to IAA (n = 5) (Figure 5M). These findings suggested that information processing of alarm pheromone does not contribute mainly to the neural activity of Class II KCs, which was observed in the brains of workers involved in the formation of the hot defensive bee ball.

Finally, we analyzed the tissue specificity of *Acks* induction under a high temperature. Quantitative RT-PCR using RNAs extracted from brain, thorax and abdomen of workers revealed that statistically significant and prominent *Acks* induction was observed only in the brain when the bees were exposed to high temperature, and scarce *Acks* expression was detected in the thorax and abdomen under both room and high temperatures (Figure 5O), further supporting that *Acks* induction under a high temperature reflects neural activity and does not result from some ‘heat shock responses’ that could occur independently of neural activity.

## Discussion

In the present study, we identified *Acks*, a Japanese honeybee homolog of *kakusei*, which is a neural IEG in the European honeybee. Although no *kakusei* homolog has been identified in any other insect species, including the fruit fly, mosquito, and moth, this finding indicates that a *kakusei* homolog is conserved at least among related but allopatric honeybee species, *A. mellifera* and *A. cerana*. The *Acks* transcript contains an additional approximately 700b sequence at the 3′-end, but overall sequence similarity with the *kakusei* transcript was conserved throughout the *Acks* transcript, including sequences corresponding to both the neural activity-inducible and constitutive-type *kakusei* transcripts (Figure 1A). In addition, expression analysis revealed that, like *kakusei*, *Acks* transcript expression levels transiently increase after seizure induced by awakening from anesthesia (Figure 1B and C), and the induced *Acks* expression can be broadly visualized in several brain regions (Figure 2). These findings suggest that the function of the *Acks* transcript is similar to that of the *kakusei* transcript as a nuclear non-coding RNA.

While the European honeybee *kakusei* showed a expression peak 15 min after seizure induction [13], the expression level of the *Acks* transcript reached a maximum 30 min after seizure induction (Figure 1B). Nonetheless, our expression analysis showed that *Acks* has the properties of a neural IEG, even under high temperature (46°C, Figure 1C), which allowed us to detect the brain regions in the Japanese worker honeybees that were active during the formation of a hot defensive bee ball.

Here, we first demonstrated that *Acks* signals were detected in the MBs, especially in the Class II KCs of workers at 30 or 60 min after the bee ball formation, and neurons located in a restricted area between the DLs and the OLs of workers at 60 min whereas they were scarcely detected at 0 min (Figure 4, Figure S3, and Figure S4), strongly suggesting that these neurons are highly activated in the brains of workers involved in forming the hot defensive bee ball.

Interestingly, the *Acks* distribution patterns observed in the brains of workers involved in the bee ball formation were clearly mimicked by those observed in the brains of heat-exposed workers but not by those observed in IAA-exposed workers (Figure 5). These results strongly suggest that the neural activity detected in the brains of workers involved in the formation of the hot defensive bee ball is induced by heat but not by IAA generated during the formation of the bee ball. There are two possible explanations for this result: 1) The neural activity detected in the brains of workers involved in the formation of a hot defensive bee ball reflects thermal stimuli processing in the brain. 2) Alternatively, the *Acks* induction is evoked as a ‘heat shock response’ in some population of brain neurons.

As for the first possibility, the MBs are believed to be a higher-order center of the insect brain that processes complex multimodal information [18], [19], [22], [23]. Sophisticated roles in adaptive behaviors such as predictive motor actions [24], short- and long-term associative memory [25]–[30], and temperature preference behavior [31], are currently ascribed to the MBs. On the other hand, the formation of a hot defensive bee ball is a highly adaptive anti-predator behavior. Because there is only 3–5°C difference in the lethal temperature between the Japanese honeybee and the giant hornet [6], [16], accurate monitoring and precise control of heat generation during forming a hot defensive bee ball seem critical for the Japanese honeybees. In insect, the mechanism of temperature sensing has been studied mainly in *Drosophila melanogaster* and is known to depend on some transient receptor potential (TRP) channels [32]–[34]. *Drosophila* TRPA1 (DTRPA1) [32], [35], [36], Pyrexia [37], and Painless [33], [38], [39], all of which are TRPA subfamily members, respond to “warm” or “hot” temperatures and are required for various temperature-related behaviors (The temperature thresholds are 24–29°C, 37.5–40°C, and 42–45°C, for DTRPA1, Pyrexia, and Painless, respectively). These thermosensitive TRPA channels are expressed in several body parts not only at the periphery but also in the brain [35], [38]. Interestingly, in the brains of adult flies, Painless is mostly expressed in the MBs [38]. Thus, it might be that the Class II KCs are involved in temperature sensing in the Japanese honeybee workers during the formation of a hot defensive bee ball. We speculate that the MBs, especially the Class II KCs, might be involved in thermal information processing, to appropriately regulate the duration of flight muscle vibration and control heat generation during forming the bee ball, although the projection pattern of the thermosensitive neurons to the MBs is not well characterized in insects [40].

Statistical analyses indicated that the *Acks* signal density decreased to approximately 45% by antennae deprivation in heat-exposed workers (Figure 5M), suggesting that there is some unknown projection(s) from the antennal thermoreceptive neurons to the Class II KCs. In contrast, quantitative RT-PCR revealed that the amount of *Acks* transcript in the whole MBs of the heat-exposed workers was not significantly affected by antennae deprivation. Therefore, it might be that the contribution of the antennal sensory input to the neural activity of the MBs of heat-exposed workers is partial or minor. It is plausible that some MB neurons that receive thermal information from peripheral neurons expressing TRP channels and/or other MB neurons expressing TRP channels themselves are responsible for the increased neural activity in the MBs of heat-exposed workers.

In the European honeybee, somata of a GABAergic recurrent neuron cluster called A3v are located in this restricted brain portion between the DLs and lobula of the OLs. These neurons receive input from the lobe of the MBs and provide inhibitory feedback to the calyces [41], [42], [43]. A previous electrophysiological study demonstrated that individual feedback neurons showed multimodal sensitivity [44]. It might be that the neurons located in this area are also involved in processing thermal information in the worker honeybees. To test this possibility, it will be necessary to examine whether the *Acks*-expressing neurons are A3v neurons by double staining for *Acks* expression and immunoreactivity to GAD, a GABA-synthesizing enzyme.

As for the above second possibility, we previously showed that the expression of the *kakusei* transcript was induced specifically in the brain but not in any other body part when the European honeybee workers woke up from anesthesia [13]. In the present study, we also showed that *Acks* was induced almost exclusively in the brain under the high temperature (Figure 5O). These results seem to argue against the possibility that *Acks* induction in the brains of workers involved in the formation of a hot defensive bee ball or heat-exposed workers represents mere ‘heat shock response’. We cannot, however, completely exclude the second possibility, because some vertebrate heat shock proteins are induced in specific body parts [45], although other heat shock protein genes are expressed ubiquitously irrespective to the body part and cell types [46]. Because some physiological processes operating in the nervous system, such as the activation and inactivation of ion channels, the conduction velocity of action potentials, presynaptic transmitter release and postsynaptic reception, are considerably affected under high temperature condition [47], [48], it could be that the neural activity detected in the heat-exposed workers reflects a direct effect of high temperature. To clarify this point, it would be necessary to isolate thermosensitive TRP channels and examine their possible involvement in forming a hot defensive bee ball.

There was no distinct *Acks* signal in the brains of IAA-exposed workers (Figure 5). This result, however, might not necessarily mean that IAA plays no significant role in this defensive behavior because the *Acks* induction was not clearly observed in the ALs of the Japanese honeybee workers even after the seizure-induction, suggesting that *Acks* might not be applicable for detection of neural activity in the ALs (data not shown).

We previously used *kakusei* to demonstrate that neural activity of the small type KCs, one subtype of the Class I KCs whose somata are located at the inner core of the calyces, is preferentially increased in the brains of European honeybee foragers [13]. In contrast, in the present study, we detected unique neural activity of the Class II KCs in the brains of Japanese honeybee workers involved in the formation of a hot defensive bee ball. These results suggest that the underlying neural mechanisms are largely different between the foraging behavior and the hot defensive bee ball formation. Although the details of such a species-specific neural mechanism remain largely unknown, our findings may provide important insight into the neural mechanisms underlying the species-specific adaptive behaviors. Future electrophysiological and comparative studies will further elucidate the neural circuits involved in this defensive behavior of the Japanese honeybee workers.

## Materials and Methods

### Bees

Japanese honeybees (*Apis cerana japonica*) maintained at the apiary in Tamagawa University (Tokyo, Japan) and giant hornets (*Vespa mandarinia japonica*) caught near the apiary were used for all experiments.

### cDNA cloning

Total RNA was extracted from the whole brains of Japanese honeybee workers with seizures induced by awakening from anesthesia, using TRIzol Reagent (Invitrogen, Carlsbad, CA). To obtain cDNA fragments of the *kakusei* homolog in Japanese honeybee, PCR was performed using a set of primers designed based on the nucleotide sequence of the European honeybee *kakusei*, 5′-GGGGAAGCCAGGAGCCGCGGGTTTACAT-3′ and 5′-AGGCAACAGCACACCATGGGCCTTGGAT-3′, with Ex Taq (Takara, Tokyo, Japan). After sequencing the PCR products, we performed the RACE method with a SMART RACE cDNA Amplification Kit (Clontech, Mountain View, CA) following the manufacturer's instruction. Amplification was performed with two sets of gene-specific primers for 5′-RACE and 3′-RACE. These amplified products were subcloned into pGEM-T vectors (Promega, Madison, WI) and sequenced.

### Quantitative RT-PCR of the *Acks* transcript in the brains of workers after seizure induction

Japanese honeybee workers kept in a plastic chamber were maintained overnight in a dark incubator (LH-70CCFL; NK System, Osaka, Japan) at 25°C. The bees were then anesthetized in CO_2_ for 3 min. To induce seizures, the bees were then left in normal air at room temperature. The bees were sampled at 0, 15, 30, 45, 60, 90, 120, 150, or 180 min after seizure induction. Control bees were maintained continuously in CO_2_ and collected at the same time points (bees at 0 min were identical to seizure induced bees at 0 min). Bees used for the high temperature experiment were anesthetized and placed in an incubator set to 46°C. We sampled the bees at the same time points as in the previous experiments under room temperature for up to 150 min after seizure induction (at 180 min, the workers died). After anesthetizing workers by immersing them in ice-cold water, we dissected two brains each from five lots of workers at each time point. After homogenizing these samples with a bead cell crusher (MS-100; Tomy, Tokyo, Japan), total RNA was extracted using TRIzol reagent. RNA was reverse transcribed with a PrimeScript RT reagent Kit (Takara) and quantitative RT-PCR was performed with LightCycler (Roche, Nutley, NJ) using SYBR Premix Ex TaqII (Takara) and gene-specific primers (*Acks*; 5′-AGTGATGTCTGACCGAGCA-3′ and 5′-CGAACGCACTTTGGTTAGTC-3′ *ef-1α*; 5′-TTGGTTTAAGGGATGGACTG-3′ and 5′-CCATACCTGGTTTCAACACA-3′ [49]). PCR products of *Acks* and *ef-1α* of known concentrations were used as standards. The amount of *Acks* transcript was normalized with that of *ef-1α* and calculated as the expression level relative to the value of the samples at 0 min in the room temperature experiment. Tukey-Kramer's test was performed to examine the significant difference in the relative expression of *Acks* among the time points using JMP software (SAS, Cary, NC). There was no significant difference in the expression of e*f-1α* between control and seizure-induced workers irrespective of experimental temperature (data not shown).

### Sampling of workers from a hot defensive bee ball

To continuously collect worker honeybees from the same bee ball, we used the following sampling procedure. A worker hornet with its stinger cut off and hung by a wire around its thorax, was presented to the bees at the entrance of the beehive. As soon as a bee ball was formed in the hive, we placed the bee ball in a beaker to separate the workers forming the bee ball from the other nestmates. The bees were collected from the surface of the bee ball with long tweezers at 0, 30, and 60 min after separation of the bee ball. We sampled the workers from four bee balls collected from three colonies. After anesthetizing the bees in ice-cold water, their brains were dissected, embedded in TissueTek O.C.T. Compound (Sakura Finetek, Torrance, CA), and rapidly frozen in dry ice and stored at −80°C until use.

### Exposure to high temperature

Japanese honeybee workers (n = 10 for each of antennae-deprived and intact workers) kept in a plastic chamber were maintained overnight in a dark incubator at 33°C. The workers were then placed into an illuminated incubator set to 46°C. After 30 min, they were anesthetized in ice-cold water and their brains were dissected. To deprive antennae, both antennae were cut with fine scissors at the base of the scapus. The brains of five to six workers for each group were used for *in situ* hybridization. To validate the tissue specificity of temperature-induced *Acks* expression, total RNA was extracted from the brains, thoraxes, and abdomens of five heat-exposed intact workers and then subjected to quantitative RT-PCR. As MB samples used for quantitative RT-PCR, the OLs, SOG and DLs were cut off from the dissected brains and the remaining MBs were used for the RNA extraction. We used three MBs each from four lots of workers for each group. Statistical analysis was conducted using JMP software. For group comparisons of two factors, a two-way ANOVA was conducted. A P value less than 0.05 was regarded as significant.

### Exposure to IAA

Japanese honeybee workers maintained in the condition similar to those used for the heat-exposure experiments were placed into a Ziplock bag (Lion, Tokyo, Japan). The workers were then presented with a filter paper, on which IAA (5 µl) was spotted, for 10 min. After the workers were removed from the Ziplock bag and left in normal air for 20 min, they were anesthetized in ice-cold water and their brains were dissected.

### *In situ* hybridization and quantification of the density of the *Acks* signals

Digoxigenin (DIG)-labeled sense or anti-sense riboprobes corresponding to +4530b to +4806b were synthesized by *in vitro* transcription with a DIG RNA Labeling Mix (Roche). Frozen coronal brain sections (10 µm thick) were fixed in 4% paraformaldehyde in phosphate buffer (PB; pH 7.4) overnight at 4°C, treated with proteinase K (10 µg/ml) for 15 min and then with HCl (0.2 N) for 10 min, followed by acetic-anhydride solution for 10 min at room temperature. The slides were washed with PB between each step. After dehydration through an ascending series of ethanol solutions, brain sections were hybridized with the riboprobes overnight at 60°C (>16 h). The riboprobes were diluted in hybridization buffer (50% formamide, 10 mM Tris-HCl, 200 µg/ml tRNA, 1×Denhardt's solution, 10% dextran sulfate, 600 mM NaCl, 0.25% SDS, 1 mM EDTA at pH 7.6), heat-denatured at 85°C for 10 min, and then added to each slide. After hybridization, slides were washed in 50% formamide and 2×SSC at 60°C for 30 min, treated with 10 µg/ml RNase A (Sigma-Aldrich, St. Louis, MO) in TNE (10 mM Tris-HCl, 1 mM EDTA, 500 mM NaCl at pH 7.6) at 37°C for 30 min, and washed at 60°C in 2×SSC for 20 min and twice in 0.2×SSC for 20 min. DIG-labeled riboprobes were detected immunocytochemically with Anti-DIG Peroxidase (1∶500; Roche), TSA Biotin System (PerkinElmer, Salem, MA), Alkaline Phosphatase Streptavidin (1∶1000; Vector Laboratories, Burlingame, CA) and NBT/BCIP stock solution (Roche) according to the manufacturer's protocol.

To quantify *Acks* signal density, we randomly selected *in situ* hybridization sections that contained the MB pedunculus and DLs (as a schematic drawing shown in Figure 2B) for each experiment. The square measure of each brain area containing *Acks*-positive neurons (specific soma area) was measured using ImageJ analysis software (NIH, http://rsb.info.nih.gov/ij). At the same time, the number of *Acks* signals in the selected area was manually counted and divided by the square measure to calculate the *Acks* signal density. The density of signals is presented as the value relative to 10,000 µm^2^. Micrographs were numbered and an investigator blind to the bee type or treatment assignment counted the signals. Statistical analyses were conducted using JMP and Excel (Microsoft) software. Data are shown as means ± standard error (SEM). We expected that *Acks* signals detected at 0 min (in bee ball formation experiment) or in control bees (in heat-exposure and IAA-exposure experiments) would reflect background level neural activity and the neural activity in the workers forming a bee ball, heat-exposed or IAA-exposed workers could be considerably increased. Therefore, one-tailed Dunnett's test was performed to examine the significance of differences compared to that at 0 min or of control bees. In the heat-exposure experiment, Student's *t*-test was also performed to compare intact workers with antennae-deprived workers.

## Supporting Information

Figure S1 **Neural activity in the middle part of the brain 30 min after the bee ball formation.** *In situ* hybridization was performed using a whole brain section, which corresponds to a middle part of a brain, of a worker collected 30 min after the bee ball formation. In this Figure, an example of the result for the whole right brain hemisphere of a worker is presented to show the overall distribution of the *Acks* signals in the brain. Note that panels showing the results of *in situ* hybridization in Figure 4, S3 and S4 are collected from some sections that are used for the same *in situ* hybridization experiments, respectively. Black arrows indicate clusters of induced *Acks* signals. The bar indicates 250 µm.
(TIFF)

Figure S2 **Neural activity in the middle part of the brain 60 min after the bee ball formation.** *In situ* hybridization was performed using a whole brain section, which corresponds to a middle part of a brain, of a worker collected 60 min after the bee ball formation. The bar indicates 250 µm.
(TIFF)

Figure S3 **Neural activity in the more rostral part of the brain during bee ball formation.** (A) Schematic diagram of the lateral view of a bee brain. The green line indicates the position of sections that correspond to a more rostral part of the brain. (B) Schematic representation of the *Acks* signals detected in the right brain hemisphere of the workers that formed a bee ball. The red dots indicate induced *Acks* signals at 30 or 60 min after the bee ball formation. The boxed regions (a–d) correspond to the Class I KCs (a), parts of the Class II KCs (b and c) and a part of the OL (d) whose *in situ* hybridization results are presented in the right panels (D–O). (C) Magnified schematic representation of the MB indicating the distribution of the somata of the Class I (green) and the Class II KCs (yellow), respectively. (D–O) *In situ* hybridization of *Acks* in each brain area shown in (B) in the brains of workers 0 (D, G, J, and M), 30 (E, H, K, and N) and 60 min (F, I, L, and O) after formation of the bee ball. (D–F), (G–I), (J–L), and (M–O) correspond to boxed brain regions (a), (b), (c), and (d), respectively. The dotted *Acks* signals were detected most densely in the Class II KCs (H, I, K, and L), and less densely in the Class I KCs (E and F) at 30 and 60 min after the bee ball formation. Note that the *Acks* signals were detected less densely in the OLs (O) at 60 min after the bee ball formation. Bars indicate 50 µm. Abbreviations and colors and dotted ellipse are as in Figure 4.
(TIFF)

Figure S4 **Neural activity in the caudal part of the brain during bee ball formation.** (A) Schematic diagram of a lateral view of a bee brain. The green line indicates the position of sections that correspond to a more caudal part of the brain. (B) Schematic representation of the *Acks* signals detected in the right brain hemisphere of the workers that formed the bee ball. The red dots indicate induced *Acks* signals at 30 or 60 min after the bee ball formation. The boxed regions (a–e) correspond to the Class I KCs (a), parts of the Class II KCs (b and c), a part of the OL (d), and the region adjacent to the OL (e), whose *in situ* hybridization results are presented in the right panels (D–R). (C) Magnified schematic representation of the MB indicating the distribution of the somata of the Class I (green) and the Class II KCs (yellow), respectively. (D–R) *In situ* hybridization of *Acks* in each brain area shown in (B) in the brains of workers at 0 (D, G, J, M, and P), 30 (E, H, K, N, and Q), and 60 min (F, I, L, O, and R) after bee ball formation. (D–F), (G–I), (J–L), (M–O), and (P–R) correspond to the boxed brain regions (a), (b), (c), (d), and (e), respectively. The dotted *Acks* signals were detected most densely in the Class II KCs (H, I, K, and L), and less densely in the Class I KCs (E and F). Note that the *Acks* signals were detected less densely in the OLs (O) at 60 min after the bee ball formation. No or scarce signal were detected in the region adjacent to the OL (P–R) irrespective of the sampling time. Bars indicate 50 µm. Abbreviations and colors and dotted ellipse are as in Figure 4.
(TIFF)
