# Supplementary figures and images for: Detection of Neural Activity in the Brains of Japanese Honeybee Workers during the Formation of a “Hot Defensive Bee Ball”
Source: PLoS One. 2012 Mar 14;7(3):e32902. doi: 10.1371/journal.pone.0032902 (PMC3303784; doi:10.1371/journal.pone.0032902)

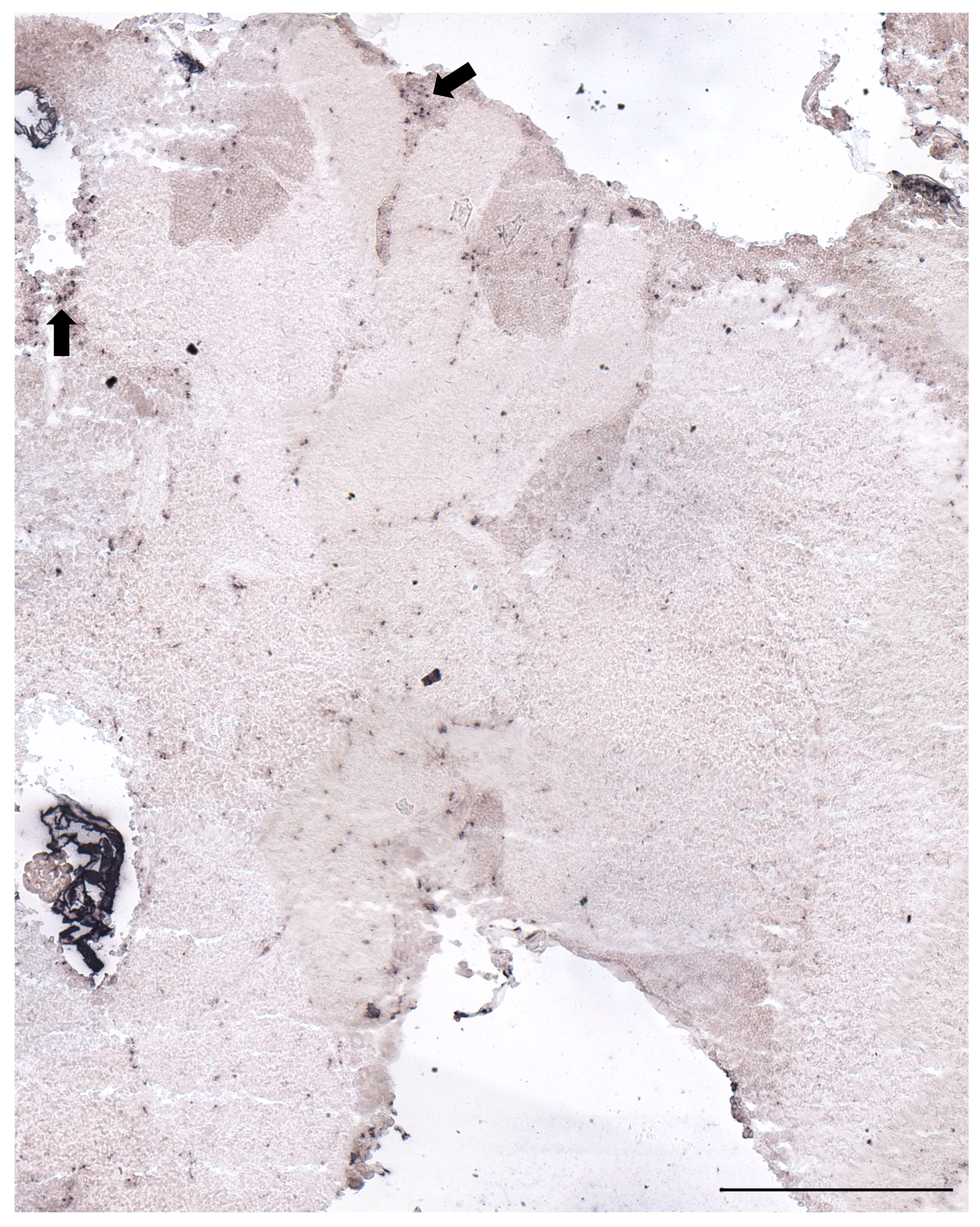

Supplement: Figure S1 — Neural activity in the middle part of the brain 30 min after the bee ball formation. In situ hybridization was performed using a whole brain section, which corresponds to a middle part of a brain, of a worker collected 30 min after the bee ball formation. In this Figure, an example of the result for the whole right brain hemisphere of a worker is presented to show the overall distribution of the Acks signals in the brain. Note that panels showing the results of in situ hybridization in Figure 4, S3 and S4 are collected from some sections that are used for the same in situ hybridization experiments, respectively. Black arrows indicate clusters of induced Acks signals. The bar indicates 250 µm. (TIFF) [file pone.0032902.s001.tiff]

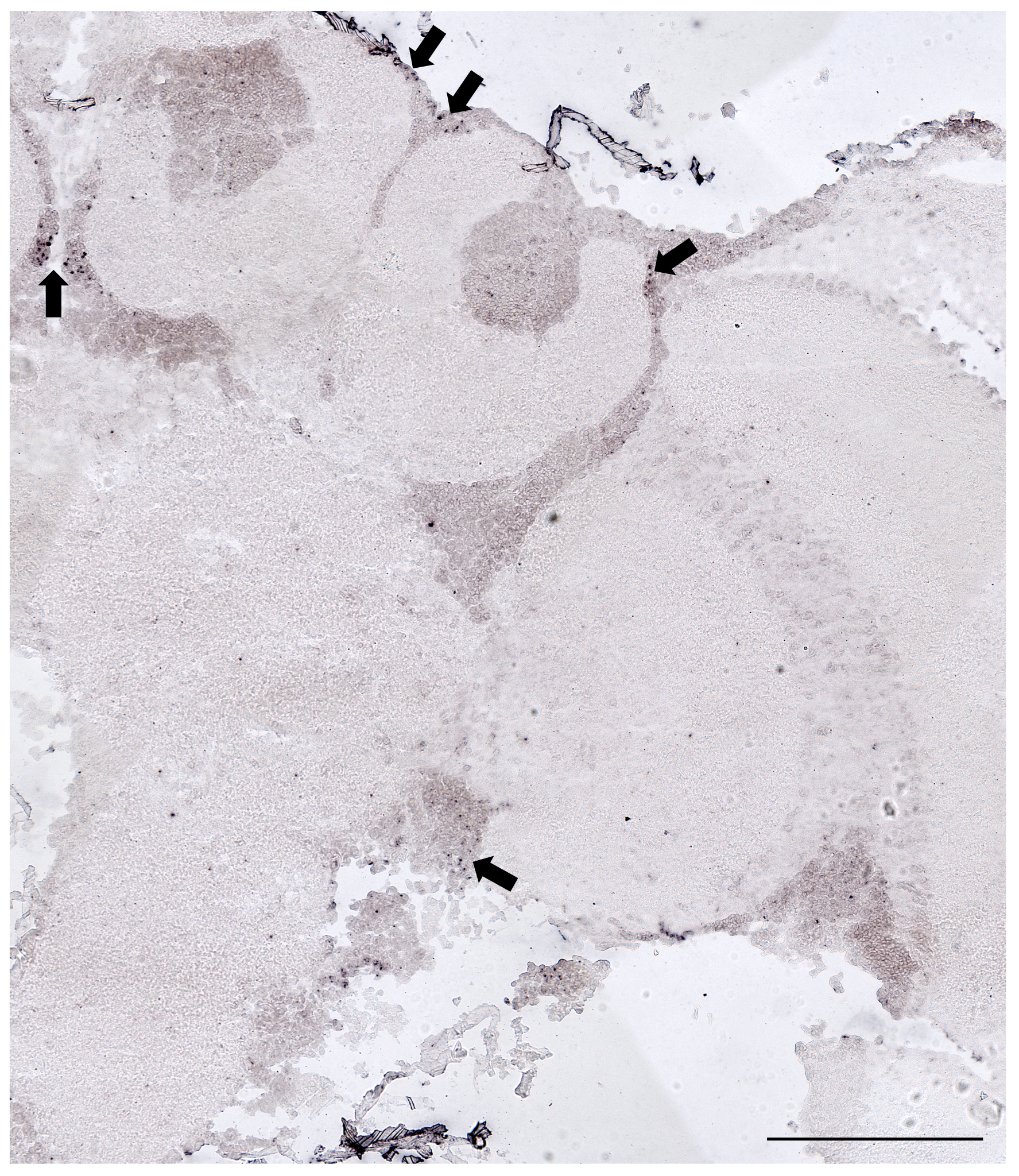

Supplement: Figure S2 — Neural activity in the middle part of the brain 60 min after the bee ball formation. In situ hybridization was performed using a whole brain section, which corresponds to a middle part of a brain, of a worker collected 60 min after the bee ball formation. The bar indicates 250 µm. (TIFF) [file pone.0032902.s002.tiff]

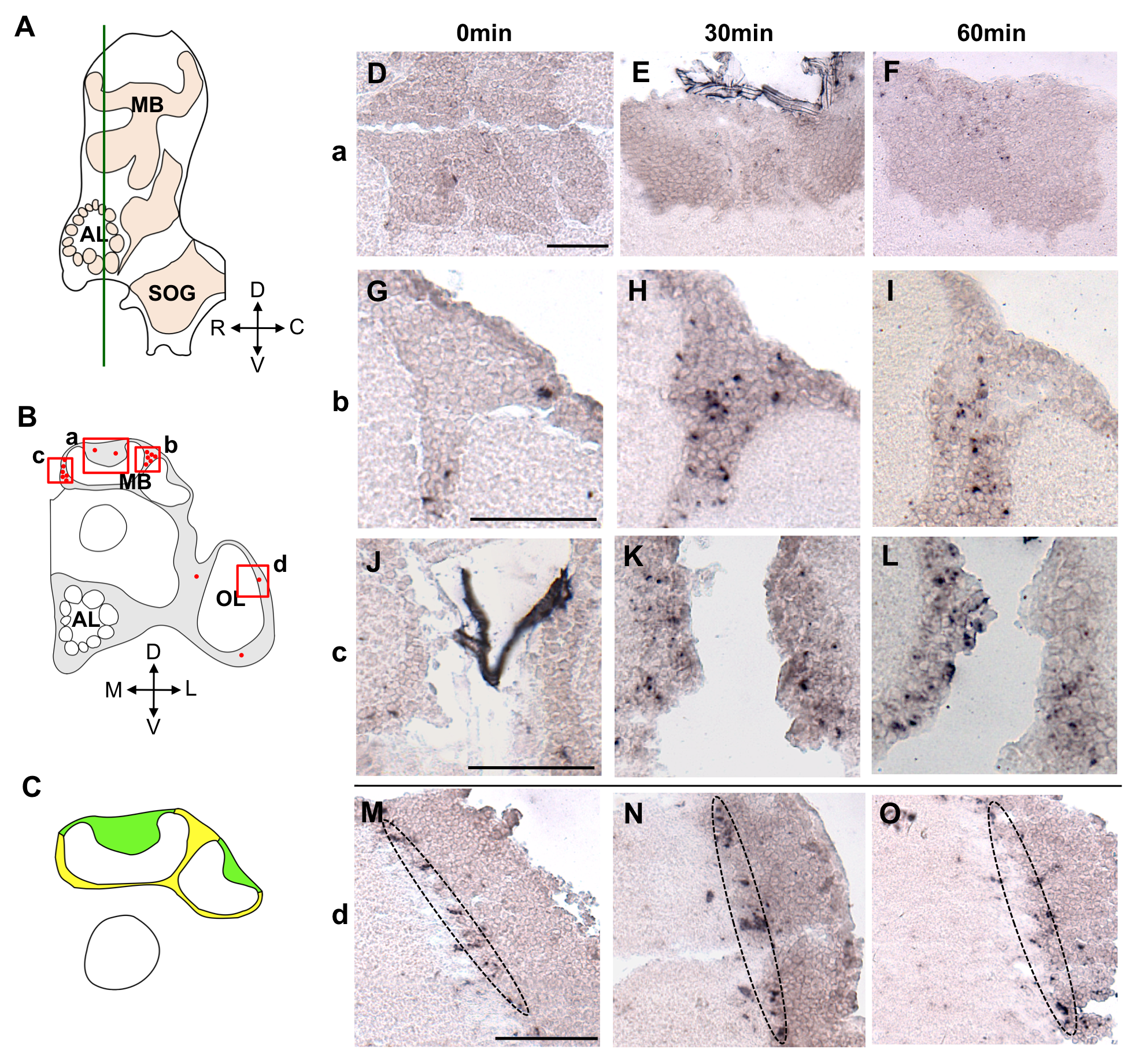

Supplement: Figure S3 — Neural activity in the more rostral part of the brain during bee ball formation. (A) Schematic diagram of the lateral view of a bee brain. The green line indicates the position of sections that correspond to a more rostral part of the brain. (B) Schematic representation of the Acks signals detected in the right brain hemisphere of the workers that formed a bee ball. The red dots indicate induced Acks signals at 30 or 60 min after the bee ball formation. The boxed regions (a–d) correspond to the Class I KCs (a), parts of the Class II KCs (b and c) and a part of the OL (d) whose in situ hybridization results are presented in the right panels (D–O). (C) Magnified schematic representation of the MB indicating the distribution of the somata of the Class I (green) and the Class II KCs (yellow), respectively. (D–O) In situ hybridization of Acks in each brain area shown in (B) in the brains of workers 0 (D, G, J, and M), 30 (E, H, K, and N) and 60 min (F, I, L, and O) after formation of the bee ball. (D–F), (G–I), (J–L), and (M–O) correspond to boxed brain regions (a), (b), (c), and (d), respectively. The dotted Acks signals were detected most densely in the Class II KCs (H, I, K, and L), and less densely in the Class I KCs (E and F) at 30 and 60 min after the bee ball formation. Note that the Acks signals were detected less densely in the OLs (O) at 60 min after the bee ball formation. Bars indicate 50 µm. Abbreviations and colors and dotted ellipse are as in Figure 4. (TIFF) [file pone.0032902.s003.tiff]

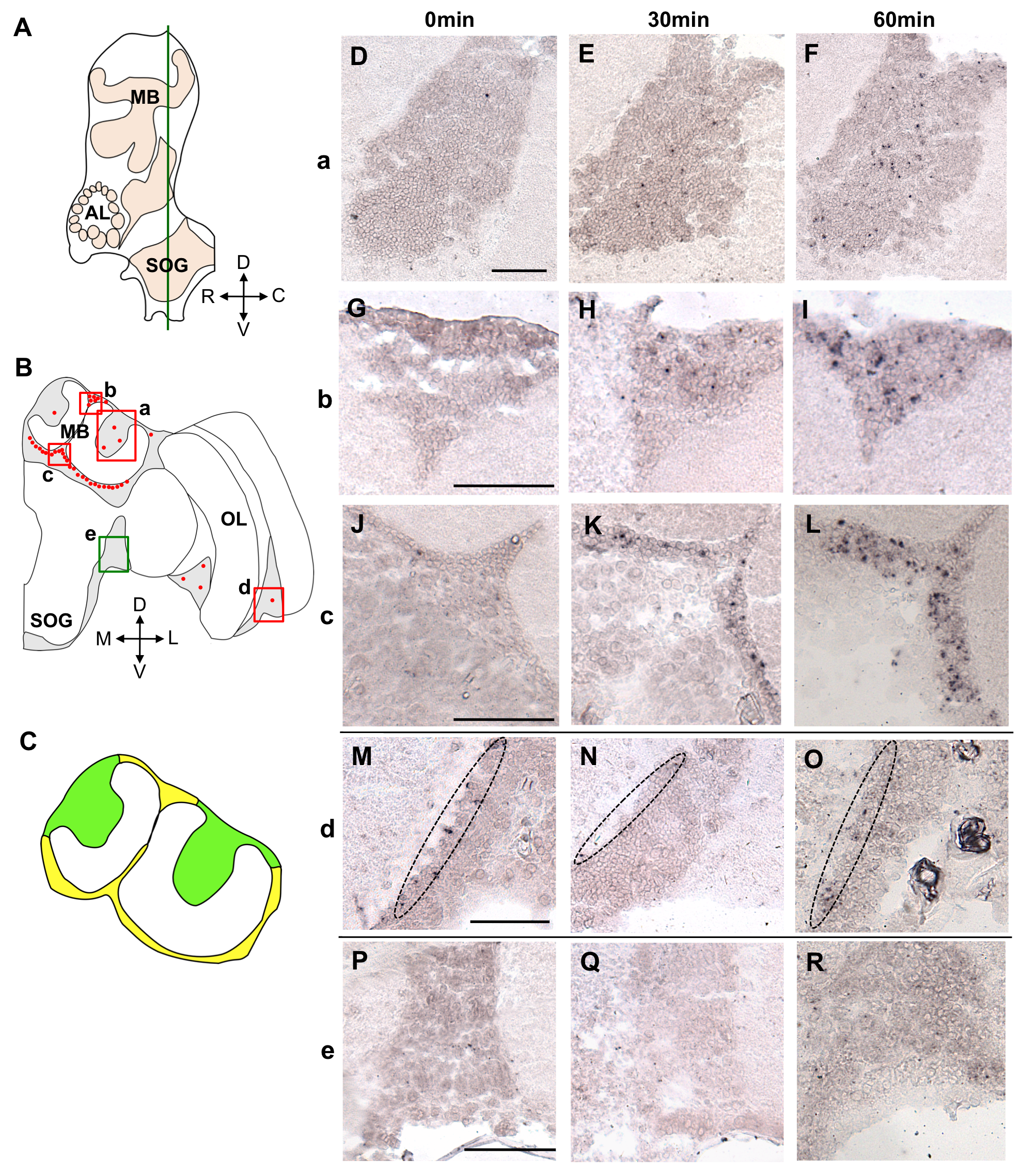

Supplement: Figure S4 — Neural activity in the caudal part of the brain during bee ball formation. (A) Schematic diagram of a lateral view of a bee brain. The green line indicates the position of sections that correspond to a more caudal part of the brain. (B) Schematic representation of the Acks signals detected in the right brain hemisphere of the workers that formed the bee ball. The red dots indicate induced Acks signals at 30 or 60 min after the bee ball formation. The boxed regions (a–e) correspond to the Class I KCs (a), parts of the Class II KCs (b and c), a part of the OL (d), and the region adjacent to the OL (e), whose in situ hybridization results are presented in the right panels (D–R). (C) Magnified schematic representation of the MB indicating the distribution of the somata of the Class I (green) and the Class II KCs (yellow), respectively. (D–R) In situ hybridization of Acks in each brain area shown in (B) in the brains of workers at 0 (D, G, J, M, and P), 30 (E, H, K, N, and Q), and 60 min (F, I, L, O, and R) after bee ball formation. (D–F), (G–I), (J–L), (M–O), and (P–R) correspond to the boxed brain regions (a), (b), (c), (d), and (e), respectively. The dotted Acks signals were detected most densely in the Class II KCs (H, I, K, and L), and less densely in the Class I KCs (E and F). Note that the Acks signals were detected less densely in the OLs (O) at 60 min after the bee ball formation. No or scarce signal were detected in the region adjacent to the OL (P–R) irrespective of the sampling time. Bars indicate 50 µm. Abbreviations and colors and dotted ellipse are as in Figure 4. (TIFF) [file pone.0032902.s004.tiff]
